# Bactericidal activity of oxacillin and glycopeptides against *Staphylococcus aureus *in patients with endocarditis: Looking for a relationship between tolerance and outcome

**DOI:** 10.1186/1476-0711-10-26

**Published:** 2011-06-09

**Authors:** Maria Bruna Pasticci, Amedeo Moretti, Giuliano Stagni, Veronica Ravasio, Laura Soavi, Annibale Raglio, Francesca Vailati, Angela Cardaccia, Antonella Santucci, Rita Papili, Alessio Sgrelli, Carlo Pallotto, Franco Baldelli

**Affiliations:** 1 Section of Infectious Diseases, Department of Experimental Medicine and Biochemical Sciences, University of Perugia, Perugia, Italy; 2 Infectious Disease Department, Ospedali Riuniti di Bergamo, Bergamo, Italy; 3 Unit of Microbiology and Virology, Ospedali Riuniti di Bergamo, Bergamo, Italy

## Abstract

**Background:**

There is no clear relationship between *in vitro *bactericidal activity tests and clinical outcome. We studied bactericidal activity of oxacillin, vancomycin and teicoplanin against *Staphylococcus aureus *isolates in patients with endocarditis and then we sought to determine if there was a relationship between *in vitro *bactericidal activity and clinical outcome.

**Methods:**

Minimal bacteriostatic and minimal bactericidal concentrations were determined for *Staphylococcus aureus *strains isolated from patients with endocarditis following standardized methods. Medical records were reviewed retrospectively to collect data on antimicrobial susceptibility at admission, antimicrobial therapy, need for surgery, embolic events and outcome.

**Results and Discussion:**

Sixty-two *Staphylococcus aureus *strains were studied in 62 patients with endocarditis. Overall, 91.9% definite, 21% methicillin resistant and 72.6% cured. Surgery was performed in 32.3% and embolic events were documented in 64.5%. Tolerance to oxacillin and teicoplanin was more common than vancomycin tolerance among methicillin susceptible *Staphylococcus aureus*. Among methicillin resistant *Staphylococcus aureus *teicoplanin was shown to have a higher rate of tolerance than vancomycin. No statistically significant differences on clinical outcome between oxacillin tolerant and oxacillin non tolerant *Staphylococcus aureus *infections were observed. Tolerance to oxacillin did not adversely affect clinical outcomes of patients with methicillin susceptible *Staphylococcus aureus *endocarditis treated with a combination of antimicrobials including oxacillin. The cure rate was significantly lower among patients with methicillin resistant *Staphylococcus aureus *endocarditis.

**Conclusions:**

In vitro bactericidal test results were not valid predictors of clinical outcome. Physicians need to use additional parameters when treating patients with staphylococcal endocarditis.

## Background

In recently published surveys*Staphylococcus aureus *(*S.aureus*) is reported to overstep viridans *Streptococci *as a cause of endocarditis (IE) and associated morbidities and mortality [1,2]. *S.aureus *is an extraordinarily adaptable bacterium, developing increasing patterns of resistance which contribute to clinical failures. Penicillin resistance was soon followed by methicillin resistance, which always includes resistance to all beta-lactam antimicrobials and often to several other classes of antibiotics [3]. The efficacy of vancomycin against methicillin resistant *S.aureus *(MRSA) has been reported to be inferior to that of beta-lactams against methicillin susceptible *S.aureus *(MSSA) due to its slower in vitro bactericidal activity with a lower clinical response [4-6]. Recently other reasons for the clinical failure of vancomycin have been indicated and include: the progressive increase of vancomycin minimum inhibitory concentrations (MICs) over time, but with values still in the susceptibility range, and the emergence of *S.aureus *showing either glycopeptide hetero-resistance, an intermediate level of resistance (GISA) or full resistance (GRSA) [7-10].

Tolerance is another form of antimicrobial resistance of *S.aureus *hypothesised to be a cause of clinical failure. Tolerance has been described for anti-staphylococcal beta-lactams but involves also glycopeptide antimicrobial agents. There have been studies reporting infections caused by tolerant strains more difficult to eradicate and antimicrobial regimens with bactericidal activity superior to that of bacteriostatic regimens in the treatment of serious *S.aureus *infections. Tolerant strains are susceptible as judged by MICs but show an increasing resistance to the killing with high minimal bactericidal concentrations (MBCs) and an MIC/MBC ratio≥32. Bactericidal activity can also be evaluated with time killing curves but, independently from the method used, there are theoretical and technical difficulties in performing these tests. Thus, highly standardised methods should be followed. To date, bactericidal activity has been regarded as a desirable characteristic in antimicrobial agents when treating patients with endocarditis, while, routine MBC testing is not recommended because of the technical difficulties associated with these tests [11-15].

The aims of this study were:

a) To determine the *in vitro *bactericidal activity of oxacillin, vancomycin and teicoplanin against *S.aureus *isolated in patients with *S.aureus *endocarditis

b) To look for a relationship between *in vitro *bactericidal activity and clinical outcome using data available from patients that had been previously enrolled in observational studies on endocarditis.

## Methods

Isolates of *S.aureus *were collected from patients with IE admitted to the Infectious Disease Departments (IDD) of Perugia and Bergamo from 1988-2009. A total of 30 strains were from Perugia and 32 from Bergamo. The initial blood isolates on the day of admission were stored at -70°C until the time of in vitro testing. MICs and MBCs were determined at the IDD of Perugia under blinded conditions according to CLSI guidelines [16-18].

1) Oxacillin: Cefoxitin disk diffusion using Muller-Hinton agar plates (bioMerieux) and 30 μg cefoxitin disk (bio-Merieux) and interpreted according to CLSI break points (16), macro-method MIC using Muller Hinton Broth (MHB), inoculum 1-5 × 10^5 ^CFU/ml, stationary phase of growth) (17) and E-test MIC according to the E-test manufacturer (AB Biodisk).

2) Vancomycin: macro-method MIC (MHB, inoculum 1-5 × 10^5 ^CFU/ml, stationary phase of growth) (17) and E-test MIC.

3) Teicoplanin: macro-method MIC (MHB, inoculum 1-5 × 10^5 ^CFU/ml, stationary phase of growth) (17), E-test MIC.

MBC was determined by sub-culturing 50 μl from each vial without a visible growth onto plates of Muller Hinton agar (MHA), after 24h of incubation at 35°C. The geometric mean of duplicate colony counts were used to determine the MBC defined as the antibiotic concentration with killing of 99.9% of the initial inoculum (18). In the presence of Eagle phenomenon, MBC was the antibiotic concentration yielding persistent killing of 99.9% of the initial inoculum.

MIC_50 _and MIC_90 _were determined by interpolation from graphs of cumulated percent strains inhibited versus MIC [19].

MSSA ATCC 29213 was used as a control strain in all these experiments.

Medical records of each patient were reviewed retrospectively to collect data on: demographic data, year of diagnosis, valve localization, hospital or community acquired infection, antimicrobial susceptibility results at the time of patient admission and antimicrobial treatment, surgery, embolic events and outcomes.

There was no research related effect for patients. Patients gave informed consent to be included in the observational protocol on endocarditis cases. The protocol was approved by the institutional ethical committee of Perugia and Bergamo. All activity was conducted in accordance with the Declaration of Helsinki, and national and institutional review board.

Associations among qualitative variables were analysed using the contingency table. The statistical significance was assessed with the Fisher exact test.

## Results

Sixty-two isolates of *S.aureus *were collected from patients with endocarditis, 58% male, 91.9% definite (DE) and 8.1% possible (PE) according to Duke criteria, 47 (75.8%) native valve endocarditis (NVE) and 15 (24.2%) prosthetic valve endocarditis (PVE), three pace-maker wire (PM) infections and 37% hospital acquired. Overall, 13/62 (21%) were caused by MRSA and 76.9 of these were hospital acquired. All the isolates were reported vancomycin and teicoplanin susceptible. Overall, 45 patients (72.6%) were cured and 17 (27.4%) died of endocarditis. Surgery was performed on 20 (32.3%) and embolic events were documented in 40 cases (64.5%) (Table 1).

**Table 1:** Epidemiology of *S.aureus *endocarditis

Year	Total	DE (%)	NVE (%)	PVE (%)	PM	MRSA (%)	HA (%)	SUR (%)	EMB (%)	Cured (%)
≤2000	4	4	3	1	0	0	0	2	4	3

2001	3	3	3	0	0	1	2	3	2	3

2002	1	1	1	0	0	0	0	0	1	1

2003	1	1	1	0	0	1	1	0	1	0

2004	5	5	4	1	2	0	1	2	1	4

2005	14	14	12	2	0	2	5	5	9	11

2006	12	11	9	3	1	3	7	6	10	10

2007	3	3	2	1	0	0	1	0	2	0

2008	5	4	2	3	0	2	2	1	2	4

2009	14	11	10	4	0	4	4	1	8	9

										

Total	62	57 (91.9)	47 (75.8)	15 (24.2)	3	13 (21.0)	23 (37.0)	20 (32.3)	40 (64.5)	45 (72.6)

DE (definite endocarditis, NVE (native valve endocarditis), PVE (prosthetic valve endocarditis), PM (pace maker), HA (hospital acquired), SUR (surgery), EMB (embolism)

Oxacillin, vancomycin and teicoplanin susceptibility of 62 *S.aureus *isolates are reported in Table 2. Oxacillin and teicoplanin results in this study were in agreement with the laboratory results obtained at admission. Vancomycin susceptibility was not fully confirmed for two isolates tested. One of these was cultured from a patient in Bergamo and one from Perugia; both had macro-method MICs of 4 mg/l while their MICs with the E-test were 2.5 mg/l and 2.0 mg/l respectively.

**Table 2:** Bacteriostatic activity of oxacillin and glycopeptides

Antibiotics	Macromethod MICs N.susceptible/N.tested (%)	E-test MICs N.susceptible/N.tested (%)
□Oxacillin (MIC: S≤2 mg/l) (DISK:S≥22 mm)	49/62 (79.0%)	49/62 (79.0%)

*/**Vancomycin (S≤2 mg/l)	60/62 (96.7%)	61/62 (98.3%)

*Teicoplanin (S≤8 mg/l)	62/62 (100%)	62/62 (100%)

**Teicoplanin (S≤2 mg/l)	60/62 (96.7%)	60/62 (96.7%)

□ CLSI and EUCAST susceptibility break point, cefoxitin disk diffusion same results as oxacillin

*CLSI susceptibility break point

**EUCAST susceptibility break point

Methicillin resistance was confirmed in 13 out of 62 (21%) isolates.

**MSSA susceptibility tests: **oxacillin geometric mean MIC 0.52 mg/l (range 0.25-1), MIC_50 _0.35 mg/l, MIC_90 _0.48 mg/l, tolerance rate 63.2%; vancomycin mean MIC 1.69 mg/l (range 1-2), MIC_50 _1.1 mg/l, MIC_90 _1.7 mg/l, tolerance rate 18.4%; teicoplanin mean MIC 1.39 mg/l (range 1-2), MIC_50 _0.9 mg/l, MIC_90 _1.7 mg/l, tolerance rate 61.2%.

**MRSA susceptibility tests: **vancomycin mean MIC 2.3 mg/l (range 2-4), MIC_50 _1.5 mg/l, MIC_90 _2.5 mg/l, tolerance rate 30.8%; teicoplanin mean MIC 2.1 mg/l (range 1-4), MIC_50 _1.3 mg/l, MIC_90 _2.5 mg/l, tolerance rate 76.9% (Table 3).

**Table 3:** MIC geometric mean, MIC_50_, MIC_90 _and rate of tolerance (macromethod)

	Oxacillin				Vancomycin				Teicoplanin			
	**Mean MIC (mg/l) (range)**	**MIC **_**50 **_**(mg/l)**	**MIC**_**90 **_**(mg/l)**	**N.tolerant (%)**	**Mean MIC (mg/l) (range)**	**MIC **_**50 **_**(mg/l)**	**MIC**_**90 **_**(mg/l)**	**N. tolerant (%)**	**Mean MIC (mg/l) (range)**	**MIC **_**50 **_**(mg/l)**	**MIC**_**90 **_**(mg/l)**	**N. tolerant (%)**

MSSA N.49 (79%)	0.52 (0.25-1)	0.35	0.48	31/49 (63.2%)	1.69 (1-2)	1.1	1.7	9/49 (18.4%)	1.39 (1-2)	0.9	1.7	30/49 (61.2%)

MRSA N.13 (21%)	NA	NA	NA	NA	2.3 (2-4)	1.5	2.5	4/13 (30.8%)	2.1 (1-4)	1.3	2.5	10/13 (76.9%)

Among the 31 oxacillin tolerant strains, 8 (26%) were also vancomycin tolerant and 18 (58%) were teicoplanin tolerant. Only one of the oxacillin non tolerant isolates was vancomycin tolerant while 12 (66%) were tolerant to teicoplanin.

Antimicrobial susceptibility tests had been performed at local laboratories at the time of diagnosis, as well, treatment had been decided by the curing physicians on the basis of available susceptibility results and the clinical conditions of patients. MSSA IE antimicrobial therapy consisted of: oxacillin 37, cefazolin 5, vancomycin 4, teicoplanin 1 and not known 2. MRSA IE (13 cases) were treated with vancomycin 10, teicoplanin 1 and in 2 therapy was not known. Beta-lactam or glycopeptide antimicrobials were administered in combination with rifampin or an aminoglycoside, or a quinolone or a combination of two or more of these antimicrobials for MSSA and MRSA in most of the cases. Need for surgery had been individualised following international indications. Overall, the cure rate was 79.6% for MSSA: 31 oxacillin, 4 cefazolin, 3 vancomycin and 1 teicoplanin while the cure rate for MRSA was 46.2%: 5 vancomycin and 1 unreported treatment (p < 0.032). There was no evidence of any differences with regard to need for surgery or embolic events between MSSA and MRSA IE (Table 4). There were only four patients with MRSA IE vancomycin tolerant and all of these had been treated with antimicrobial combinations including vancomycin and had a cure rate of 25% (Table 4).

**Table 4:** Rates of cure, surgery, and embolism in endocarditis caused by MSSA and MRSA

	Cured N. (%)	Surgery N. (%)	Embolic events N. (%)
MSSA N. 49 (79%)	39/49 *(79.6%)	16/49 ** (32.7%)	33/49 *** (67.3%)

MRSA N. 13 (21%)	6/13 *(46.2%)	4/13 ** (30.8%)	7/13 *** (53.8%)

Vancomycin tolerant N. 4/13 (30.8%) Vancomycin non tolerant N. 9/13 (69.2%)	1/4 °(25,0%) 5/9 °(55,5%)		

*p < 0.032, **p = 1, ***p = 0.51, °p = 0.55

A negative effect of oxacillin tolerance was not observed either in the entire subgroup of IE cases caused by MSSA or in the subgroup of patients with MSSA IE being treated with an antibiotic regimen containing oxacillin (Table 5).

**Table 5:** Rates of cure, surgery, and embolism in MSSA endocarditis caused by oxacillin tolerant and oxacillin non-tolerant strains

MSSA 49/62 (79%)	Cured N. (%)	Surgery N. (%)	Embolic events N. (%)
Oxacillin tolerant N. 31 (63.2%)	26/31 * (83.9%)	10/31 ** (32.3%)	21/31 ** (67.7%)

Oxacillin non tolerant N. 18 (36.8%)	13/18 * (72.2%)	6/18 ** (33.3%)	12/18 ** (66.7%)

MSSA oxacillin tolerant treated with oxacillin/total MSSA oxacillin tolerant N. 24/31 (77.4%)	21/24 °(87.5%)	-	-

MSSA oxacillin non tolerant treated with oxacillin/total MSSA oxacillin non-tolerant N. 13/18 (72.2%)	10/13 °(76.9%)	-	-

*p = 0.46, **p = 1, °p = 0.64

## Discussion

This study was designed to assess the rate of tolerance to oxacillin, vancomycin and teicoplanin among *S.aureus *isolates in patients with IE. Thereafter, we sought to determine a relationship between *in vitro *bactericidal tests and clinical outcome.

The bactericidal test results of this study, defined by MBCs, showed that vancomycin bactericidal activity was not inferior to that of oxacillin and teicoplanin against MSSA. Glycopeptide tolerance was more common among MSSA oxacillin tolerant strains but a few non tolerant MSSA oxacillin with high MBC for glycopeptides were also observed.

Among MRSA isolates, vancomycin tolerance rate was inferior to that of teicoplanin and all the 4 vancomycin tolerant isolates were also teicoplanin tolerant.

Previous papers have reported that vancomycin has *in vitro *bactericidal activity slower than that of nafcillin with more frequent clinical failures in animal models and also patients being treated with vancomycin [4-6]. Technical variables such as inoculum size, growth conditions and killing curves, instead of MBCs, may be responsible for some of the differences in the results of this study. May et al. [20] found MBCs and the killing rates comparable with some discrepancies. Lack of killing *in vitro *has also been hypothesised to be a reversible phenotypic response due to growth conditions of the test with some types of constitutional changes noted in tolerant strains [21,22]. It has been reported that most beta-lactam tolerant strains of *S.aureus *show cross tolerance to vancomycin and teicoplanin [23].

Regarding clinical outcome, 79% of IE cases in this study were caused by MSSA. In these patients, oxacillin was the most common antibiotic prescribed and it was usually given in combination with other antibiotics in both oxacillin tolerant and oxacillin non-tolerant MSSA infections. Analysis was unable to demonstrate either increased mortality or morbidity in oxacillin tolerant MSSA IE patients. Strikingly, a non-statistically significant higher cure rate was observed in patients with IE caused by oxacillin tolerant MSSA in both the entire MSSA IE group and in the MSSA subgroup treated with oxacillin.

Despite its greater *in vitro *bactericidal activity, a higher rate of clinical failures was observed among patients with MRSA IE. Among MRSA IE patients, higher rates of clinical failure were observed among patients with endocarditis caused by vancomycin tolerant MRSA strains, but there were only four patients in this group of patients to comment on.

With regard to bacteriostatic activity, methicillin resistance was confirmed in 21% of the isolates. Methicillin resistance occurred more commonly among hospital acquired infections with the exception of three cases. One of these should be classified as health care associated even though the isolate had a *mecA *cassette type V, which is a marker for methicillin-resistant community acquired infection [24] and was susceptible to all other classes of antibiotics except penicillin. This patient reported a traumatic tibia fracture, was treated with external devices and discharged from hospital. Subsequently, the patient developed osteomyelitis and endocarditis with multiple septic lung emboli and myocardial abscess. The other two cases were community acquired MRSA IE from the Bergamo cohort.

Concordant susceptibility test results were obtained also with teicoplanin.

With regard to vancomycin, there was no full agreement on the grade of susceptibility obtained at admission. Two isolates had vancomycin macro-method MICs of 4 mg/l, which are intermediate susceptible values. The obtained values were still in the susceptible range when performing the E-test, being 2 mg/l for the Perugia isolate and 2.5 mg/l for the Bergamo isolate. The former was methicillin resistant, hospital acquired, non tolerant to vancomycin but teicoplanin tolerant, cultured in early prosthetic infection with valve abscess. The patient treatment included teicoplanin, followed by daptomycin and rifampin then tigecycline. The patient died. The latter isolate was methicillin resistant, hospital acquired and vancomycin tolerant, cultured from a case of pace-maker infection. The patient underwent both the surgical removal of the device and vancomycin plus fosfomicin and co-trimoxazole treatment with cure.

In this study, all 62 isolates had slightly higher macro-method vancomycin MICs than with the E-test. Previously in literature, higher vancomycin MICs have been observed with the E-test compared to the micro-dilution method. Nonetheless, the micro-dilution method and E-test both have been reported to perform differently than disk diffusion and some automated systems [25,26]. Given this, while these in vitro vancomycin susceptibility testing parameters are being standardised, it may be advisable to assess vancomycin MICs with more than one method and to carry out a close clinical and microbiological follow up while the patients are being treated with vancomycin.

A progressive increase in vancomycin MICs over time was not observed in this study [7,25,26] however, there were very few strains included before the year 2004 and all of these, though methicillin susceptible, already had vancomycin MIC of 1-2 mg/l.

## Conclusions

This study reports that oxacillin and teicoplanin tolerance was common among *S.aureus *and more common than vancomycin. This study was unable to show a statistically significant correlation between bactericidal activity *in vitro *and clinical outcome. However, one must consider that these results were from a retrospective analysis and treatment was not standardized. Additional, prospective standardized studies are needed to evaluate if *in vitro *bactericidal tests are valid predictors of clinical outcome in staphylococcal endocarditis.

## Competing interests

The authors declare that they have no competing interests.

## Authors' contributions

PMB designed the study, supervised laboratory experiments, performed clinical examinations, recruited patients, analysed the data and drafted the manuscript. AM and AC collected bacterial isolates, performed on admission susceptibility tests and laboratory experiments. VR, LS, AS and CP carried out clinical examinations and the recruitment of patients. AR and AG collected strains and performed on admission susceptibility tests. AS and RP analysed the data. GS and FB revised the manuscript. All authors read and approved the final manuscript.
